# Mitral annular disjunction: A case series and review of the literature

**DOI:** 10.3389/fcvm.2022.976066

**Published:** 2022-08-12

**Authors:** Stephanie Wu, Robert J. Siegel

**Affiliations:** Cedars-Sinai Medical Center, Smidt Heart Institute, Los Angeles, CA, United States

**Keywords:** mitral valve prolapse, mitral annular disjunction, sudden cardiac death, ventricular arrhythmia, transthoracic echocardiogram

## Abstract

Mitral annular disjunction (MAD) is an abnormal displacement of the mitral valve leaflet onto the left atrial wall and is commonly found in patients with mitral valve prolapse (MVP). The diagnosis is usually made by transthoracic echocardiography (TTE) although findings can be subtle and further cardiac imaging may be necessary. MAD has been associated with a risk of malignant ventricular arrhythmias and sudden cardiac death, therefore recognition of this diagnosis and risk stratification are highly important. In this review, we will discuss the diagnosis, clinical implications, risk stratification and management of MAD based upon currently available literature, as well as provide a series of cases showing the heterogeneity in presentation and our experience with management of this rare but potentially fatal entity.

## Introduction

Mitral valve prolapse (MVP) is a common condition that affects up to 3% of the population and is the most frequent cause of primary or degenerative mitral regurgitation (MR) (1, 2). The clinical outcome of MVP is generally related to the consequence of MR progression, however a small minority of patients with MVP are at risk for malignant arrhythmias and sudden cardiac death (SCD) (3–5). Mitral annular disjunction (MAD) is a common finding in MVP and has been associated with arrhythmias and SCD (6, 7). MAD was first described by Bharati et al. (8), and is characterized by an anomalous attachment of the posterior leaflet of the mitral valve (MV) directly onto the left atrial wall, with separation between the MV attachment and the atrium-MV junction (8–10).

MAD is a common finding in patients with concomitant MV disease, and has been reported in over 30% of patients with MVP, although this rate varies based upon different populations and imaging modalities (7, 9, 11, 12). It is unclear whether MAD precedes and potentially causes MVP, is a byproduct of MVP, or is entirely independent of MVP (13). The clinical significance of MAD in the absence of mitral valve disease is unknown, although it has been shown that the prevalence of ventricular arrhythmias in MAD with concomitant MVP and isolated MAD was not significantly different, which suggests the arrhythmogenicity of MAD alone (12, 14). The proposed mechanism of arrhythmias in MAD is related to a combination of papillary muscle fibrosis and mechanical stretch of the myocardium, which has been supported by cardiac magnetic resonance (CMR) imaging findings (3, 14, 15).

In this review, we summarize the diagnosis, clinical implications, risk stratification and management of MAD and provide a case series to show the heterogeneity in presentation of MAD.

## Mitral valve annulus anatomy and annular disjunction

The mitral annulus is a complex D-shaped structure with an anterior straight component and a posterior curved component. The anterior annulus is composed of the anterior mitral leaflet which inserts onto a band of connective tissue called the mitral-aortic curtain. The posterior mitral annulus is composed of the left atrial wall, the leaflet hinge line, the LV free wall and epicardial adipose tissue, held together by a fibrous cord. The posterior mitral leaflet hinges to the junction of the atrial and ventricular myocardium (10, 16). In MAD, the hinge line of the posterior leaflet is dislocated superiorly toward the left atrial wall and away from the left ventricular (LV) myocardium, leaving a separation between the atrial-valve junction and LV wall. Normal mitral annulus motion is coupled to LV deformation and the annulus contracts and deepens its saddle shape during ventricular systole (9, 17, 18). The disjunctive mitral annulus follows the motion of the left atrial wall instead and displays paradoxical systolic expansion and increase in mitral annulus diameter, which may have important functional implications (9, 19).

Barlow's disease is a form of degenerative MV disease characterized by myxomatous thickening, billowing and prolapse of the mitral leaflets and pronounced annular dilation, often leading to significant MR (20). The association between Barlow's disease and MAD was first described more than two decades ago, and since then multiple studies have demonstrated a high prevalence of MAD in the setting of Barlow's disease (19, 21, 22). Lee et al. showed that MAD was associated with more diffuse myxomatous MV disease with more severe mitral leaflet deformity and larger leaflet areas and billow height and volume (9). The severity of myxomatous MV disease including leaflet thickening and redundancy has been proposed as a risk factor for malignant arrhythmias and sudden cardiac death (23). In clinical practice, MV disease is often characterized by the presence and type of MVP (single versus bileaflet prolapse), however a more comprehensive approach including the evaluation of leaflet thickening and redundancy may also be important (24). Recognition of myxomatous mitral valve disease should prompt careful detection of MAD and further risk stratification, as described in the next sections.

## Diagnosis and multimodality imaging

The diagnosis of MAD is made with cardiac imaging which can be done by transthoracic echocardiography (TTE), transesophageal echocardiography (TEE), cardiac computed tomography (CT) or cardiac magnetic resonance imaging (CMR). It is defined as the absence of myocardium during systole between the MV annulus and the adjacent segment of the left ventricular wall, although MAD has not been consistently defined in prior studies or guidelines and a reference standard imaging technique has not yet been established (25). Carmo et al. described the recognition of MAD by TTE using the length of the annular disjunction during end-systole on parasternal long axis view, which was defined as the measurement from the junction of the left atrial wall and MV posterior leaflet to the top of the LV posterior wall (19). MAD has also been described based upon intraoperative TEE, and was defined as a separation between the P2 insertion into the left atrial wall and the atrial/ventricular attachment performed in a 4-chamber mid-esophageal view (22).

Although MAD can be detected by TTE and TEE, it is only visualized during ventricular systole and may be missed and underreported especially in the absence of concomitant mitral valve disease. Recognition of MAD is particularly important given its association with life-threatening arrhythmic events and should especially be investigated in those with concomitant MVP or myxomatous MV disease with arrhythmias or symptoms of arrhythmias, therefore a multi-imaging modality approach may be necessary to improve detection. Imaging tools with superior spatial resolution such as cardiac CT or CMR may serve as complementary diagnostic tools for those with a lesser degree of MAD (25, 26). Detection of MAD by echocardiography is generally evaluated with a single plane image, which can overlook disjunction without the addition of three-dimensional imaging. Cardiac CT has high spatial resolution with a wide field of view and can evaluate the mitral valve using a multiplanar reconstruction method, thereby providing detailed anatomic information, visualization of the entire circumference of the mitral valve attachment and more sensitive detection of disjunction (26, 27). CMR is considered the gold standard imaging technique for evaluating myocardial function, quantifying chambers and detecting scar (28). CMR has been shown to have more optimal detection of MAD compared to TTE and can provide risk stratification and prognostic information. Therefore CMR may be an important adjunct to echocardiography as it can better define more subtle MAD and detect markers of arrhythmia risk (25, 29).

## Clinical implications

The clinical outcome of MVP is variable and related to complications such as mitral regurgitation, congestive heart failure, infective endocarditis and stroke. The risk of malignant arrhythmias and SCD is low, occurring in ~0.2–0.4% of patients with MVP per year, and MVP is not routinely considered as a major cause of SCD (4, 30, 31). Several risk factors are known to increase the risk of life-threatening arrhythmias in patients with MVP, including female sex, higher burden of ventricular ectopy (VE), bileaflet myxomatous MV degeneration, MR severity, and flail leaflet (32–35). MAD, which is a common finding in MVP and can also occur in the absence of mitral valve disease, has been shown by multiple studies to be associated with ventricular arrhythmias. Younger age, higher burden of VE, longer longitudinal distance of MAD, and evidence of papillary muscle fibrosis on CMR have been shown to be predictors of arrhythmias in MAD (14, 36).

Dejgaard et al. showed a high prevalence of life-threatening arrhythmic events occurred in patients with MAD, with or without MVP, suggesting that the arrhythmogenesis of MAD may be independent of MVP (14). In a long-term follow-up study of 595 MVP patients, Essayagh et al. showed that while the presence of MAD was independently associated with a higher risk of arrhythmic events, this risk is progressive and delayed, likely related to progressive mitral apparatus fibrosis. The presence of MAD with MVP was not associated with increased mortality over 10 years, however the presence of arrhythmias was associated with a delayed increase in mortality from time of arrhythmia diagnosis (7). In addition to arrhythmia risk, the presence of MAD has been shown to be associated with more severe mitral leaflet and chordae tendineae deformity and LV enlargement, and MR severity correlates with the degree of MAD. These findings suggest that MAD may contribute to the progression of MR and propose that the presence of MAD may predispose future prolapse in patients without concomitant MV disease (7, 9).

## Cases

### Case 1

A 34 year old female with no past medical history who presented with sudden cardiac arrest due to ventricular fibrillation. While return of spontaneous circulation was obtained, patient developed significant cardiogenic shock requiring veno-arterial extracorporeal membrane oxygenation. A transthoracic echocardiogram (TTE) on presentation showed severely depressed left ventricular ejection fraction (LVEF) and coronary angiography excluded obstructive coronary artery disease (CAD). Her LVEF improved over time and she was taken off mechanical circulatory support. Repeat TTE showed improved LV function and evidence of bileaflet MVP, moderate mitral regurgitation (MR) and MAD, not clearly visualized on initial imaging. CMR confirmed these findings and additionally showed diffuse myocardial fibrosis and late gadolinium enhancement (LGE) in the inferolateral wall. Patient was discharged after receiving a secondary prevention implantable cardioverter defibrillator (ICD).

### Case 2

**Figure 1:**
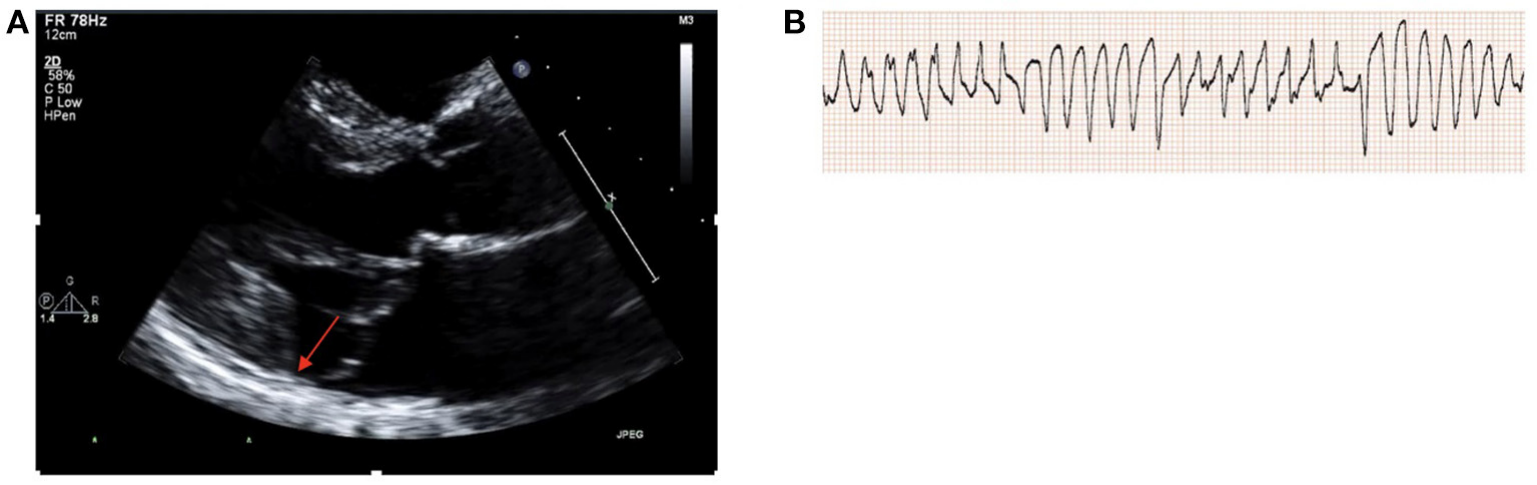
**(A)** TTE parasternal long axis demonstrating bileaflet MV prolapse and MAD (red arrow). **(B)** Induction of polymorphic ventricular tachycardia during electrophysiology study.

47 year old female with a history of MVP who presented with a syncopal episode. Ischemic work-up was negative for CAD by coronary CT angiography and TTE showed normal LVEF with bileaflet MVP, moderate MR and MAD (Figure 1A), with mild hypokinesis of the inferolateral wall and LGE seen in the inferior and inferolateral walls on CMR. Given concern for an arrhythmogenic cause of syncope, an electrophysiology study was performed with induction of polymorphic ventricular tachycardia (Figure 1B) and subsequently an ICD was implanted. On follow-up, she is on a low dose beta-blocker and has not had further significant arrhythmias and MR severity has remained stable with medical management.

### Case 3

**Figure 2:**
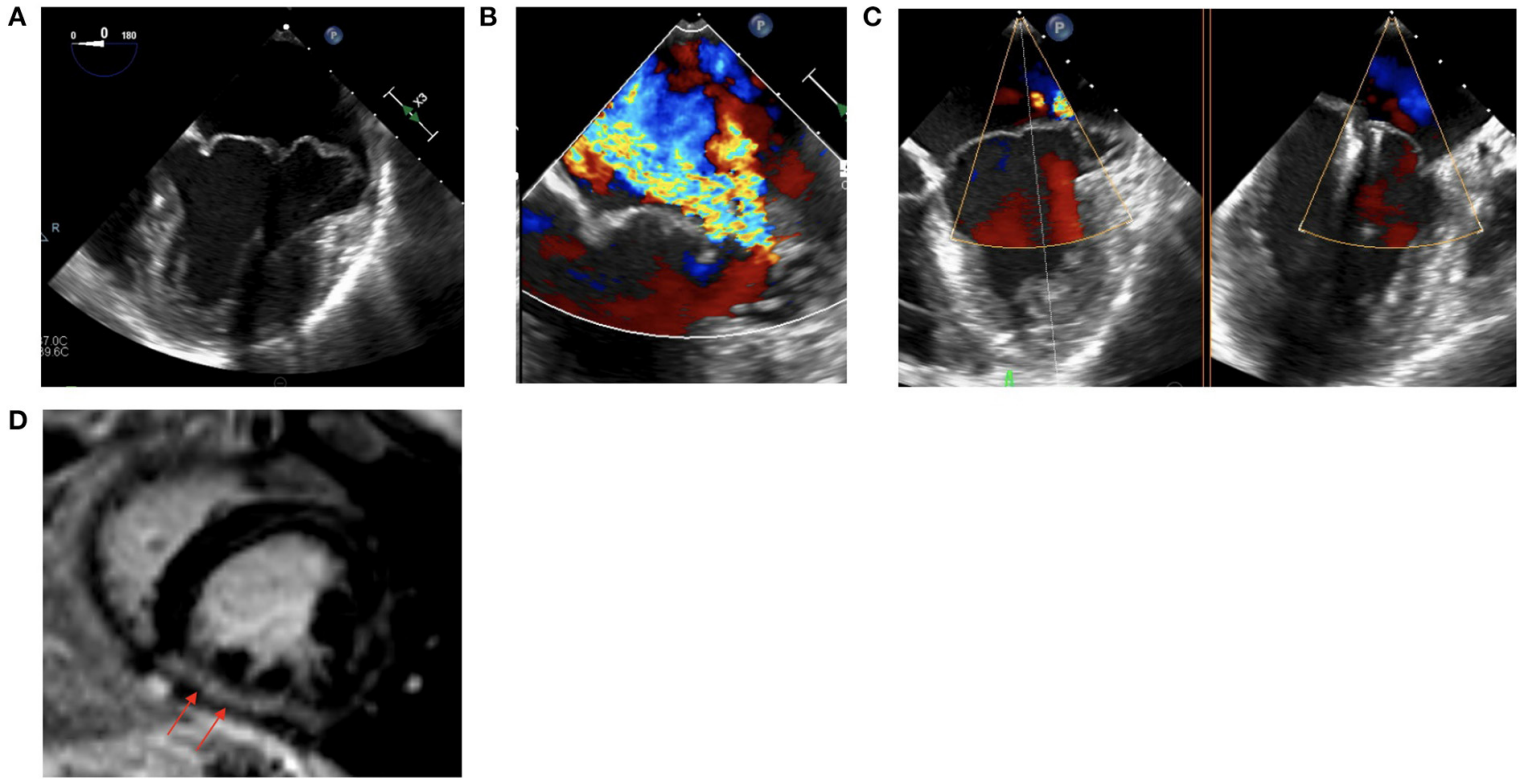
**(A)** Intraprocedural TEE showing a myxomatous MV with bileaflet prolapse and MAD. **(B)** Intraprocedural TEE showing severe MR. **(C)** Status post placement of two MitraClips with improved MR. **(D)** CMR demonstrating mid-inferior wall LGE.

49 year old female with MVP and myxomatous mitral valve disease, MAD and palpitations who presented for outpatient evaluation valvular disease. A TEE and stress echocardiogram were performed which confirmed severe MR and showed a lack of contractile reserve with peak exercise (Figures 2A,B). CMR showed a mildly reduced LVEF with presence of diffuse myocardial fibrosis and LGE in the mid-inferior wall (Figure 2D). Additionally a cardiac event monitor demonstrated 188 runs of non-sustained ventricular tachycardia as well as a 14.5% burden of VE. Given these findings, a decision was made to proceed with intervention however to avoid surgery given her mildly reduced LVEF with lack of contractile reserve, diffuse myocardial fibrosis and anatomy of her MV leaflets. Patient underwent percutaneous MV repair with two MitraClips, with significant improvement in MR (Figure 2C). Post-procedure, patient had a repeat cardiac event monitor performed which showed a significant decrease in ventricular arrhythmias and VE burden (from 14.5 to 1.2%).

### Case 4

35 year old female with a history of MVP and MR who presented for outpatient follow-up of her valvular disease. A TTE performed showed severe bileaflet prolapse and MAD with mild-moderate MR and no fibrosis or LGE was seen on CMR. Cardiac event monitoring showed rare VE with no ventricular arrhythmias. Patient has remained asymptomatic without palpitations and has been followed annually in clinic without need for intervention at this time.

### Case 5

A 34 year old female with MVP with MR and palpitations who presented for evaluation of her valvular disease. TTE showed normal LVEF, moderate bileaflet MVP with MAD, and moderate MR. No LGE was seen on CMR however ECV was elevated consistent with myocardial fibrosis. Cardiac event monitoring showed 1 run of non-sustained VT and 8.1% VE burden. Patient was referred for an electrophysiology consult, and was started on a low dose beta-blocker with recommendations for annual cardiac event monitoring in addition to TTE monitoring.

Our experience with MAD highlights the highly variable clinical course that patients may present with, from asymptomatic MVP to sudden cardiac arrest requiring mechanical circulatory support (Table 1). Case 1 and 2 emphasize the importance of MVP and MAD as potential etiologies of sudden cardiac arrest and syncope, which are often not routinely considered in the work-up of these presentations. Careful review of the echocardiogram with additional imaging as needed to delineate the mitral valve anatomy is crucial. Case 1 shows the difficulty of diagnosing MAD and the frequency with which it can be missed on routine transthoracic echocardiogram, necessitating CMR or other imaging modalities with increased spatial resolution. The current AHA/ACC/HRS guidelines recognize mitral valve prolapse as a cause of SCD but do not have specific recommendations for cardiac arrest in the setting of MVP/MAD and recommend ICD for secondary prevention after sudden cardiac arrest or hemodynamically unstable VT or sustained VT (class I) or syncope (class IIa) (37). There is a lack of data on the risk of recurrent cardiac events and use of medical therapy in this population. An invasive electrophysiology (EP) study, as performed in case 2, may be warranted in patients with abnormal imaging and high risk features to help guide risk stratification and therapy, although the usefulness is yet to be established (3, 38).

**Table 1:** Summary table of clinical features of 5 MAD patients.

	**Presentation**	**Arrhythmias**	**Valvular disease**	**CMR findings**	**Management**
Patient 1	Sudden cardiac arrest	VF arrest	Bileaflet MVP, moderate MR	Diffuse myocardial fibrosis, LGE inferolateral wall	ICD placement
Patient 2	Syncope	Inducible VT on EP study	Bileaflet MVP, moderate MR	LGE inferior and inferolateral	ICD placement
Patient 3	Follow-up known valvular disease	14.5% VE burden on cardiac event monitor	Myxomatous MV disease, severe MR	Reduced LVEF, diffuse myocardial fibrosis, LGE mid-inferior wall	Percutaneous mitral valve repair with significant decrease in VE burden
Patient 4	Follow-up known valvular disease	Rare VE	Bileaflet MVP, mild-moderate MR	Not performed	Medical management
Patient 5	Follow-up known valvular disease	8.1% VE burden and non-sustained VT on cardiac event monitor	Bileaflet MVP, moderate MR	Myocardial fibrosis, no LGE	Referred for EP consult, medical management

EP, electrophysiology; LGE, late gadolinium enhancement; LVEF, left ventricular ejection fraction; MR, mitral regurgitation; MVP, mitral valve prolapse; VE, ventricular ectopy; VF, ventricular fibrillation; VT, ventricular tachycardia.

## Risk stratification

In asymptomatic patients with MVP and MAD, risk stratification is important. Cases 3-5 reflect our routine use of ambulatory cardiac rhythm monitoring and CMR in symptomatic and asymptomatic patients. Basso et al. and Chakrabarti et al. provide clinical approaches to risk stratifying patients based upon clinical, imaging, electrocardiogram and cardiac event monitor factors (3, 38). In patients with MAD, rhythm monitoring is useful as a part of the initial diagnostic work-up and continued routine rhythm surveillance may be necessary as MAD is an independent risk factor for arrhythmias (7). In addition to assessing for the presence of non-sustained VT, studies have shown that VE originating from the papillary muscles is associated with ventricular fibrillation (39, 40). In patients with risk factors for malignant arrhythmias, evaluation with CMR or an electrophysiology study can be helpful for further risk stratification. CMR is an important method for evaluating cardiac and valvular structure and function as well as for assessing for the presence and extent of myocardial fibrosis. LGE is more commonly found in patients with MVP, most often seen in the papillary muscles or basal inferior wall, and is associated with an increased risk of arrhythmias (41, 42). Additionally the areas of LGE correlate with scar seen in pathologic specimens as well as origins of arrhythmias identified on electrophysiology studies. Patients with arrhythmic MVP may also have more diffuse fibrosis, identified on CMR by increased extracellular volume (43, 44). Careful evaluation of patient history, mitral valve characteristics, rhythm monitoring, and CMR findings such as LGE and diffuse fibrosis are important in risk stratifying patients and determining the necessity for more invasive evaluation and therapies.

## Therapies

Patients with significant valvular disease, heart failure or arrhythmias should be treated according to standard AHA/ACC guidelines, however there is little data studying the use of medical therapy such as beta-blockers or antiarrhythmics in patients with MVP and MAD. One small study looking at 112 patients with MVP showed that antiarrhythmic therapy (including beta blockers and calcium channel blockers) was not effective in decreasing burden of VE (45).

Although there are no guidelines regarding catheter ablation in MVP and MAD, this can be performed in patients with symptomatic, drug refractory ventricular arrhythmias. Syed et al. showed that catheter ablation of clinically dominant VE foci in patients with bileaflet MVP is effective at reducing symptomatic VE and reducing ICD shocks in patients with prior cardiac arrest (46). In contrast, patients with multifocal VE may not benefit as much from catheter ablation and may require repeat ablation or ICD implantation (47).

The role of primary prevention ICD in MVP and MAD is unclear. As mentioned above, risk stratification with cardiac rhythm monitoring, electrophysiology study or CMR may be useful, however there is no data at this time to support primary prevention ICD and decisions should be made on an individual basis. Secondary prevention ICD is generally indicated in patients experiencing out-of-hospital cardiac arrest after reversible causes are excluded (3).

Surgical mitral valve repair or replacement is indicated in patients with MVP and severe MR who are symptomatic or asymptomatic who meet certain echocardiographic criteria (48). While there are no specific guidelines to suggest mitral valve surgery for the treatment of ventricular arrhythmias associated with MVP, prior studies have shown a reduction in arrhythmia burden after surgery even in the absence of significant valve regurgitation, though these were small studies with few patients (49–51). Essayagh et al. showed that in 186 MAD patients, MAD remained strongly associated with arrhythmias under medical management, however the association was weaker after surgical management (7). In a recent study using three-dimensional TEE to evaluate MAD and its implications for surgical repair, the authors investigated the feasibility of successful valve repair in the setting of MVP with MAD. They showed that MAD typically occurs in patients with severe myxomatous MVP and causes alteration of annular dynamics, which resolve after appropriate annuloplasty ring suture to the ventricular myocardium, with residual MAD seen in only a small percentage of patients. This finding demonstrates the feasibility of mitral repair in the setting of MAD, and also highlights (13).

Mitral transcatheter edge-to-edge repair (TEER) with the MitraClip device was approved in the United States in 2013 for the treatment of primary MR. Surgical mitral valve repair can repair MAD in almost all patients likely due to the annuloplasty ring suture joining the mitral annulus to the LV myocardium and bridging the MAD gap, however this structural correction does not occur with TEER (7, 13, 22). Additionally, as mentioned above, although data on a large cohort of patients is lacking, smaller studies have shown improvement in ventricular arrhythmias after mitral valve surgery for MVP. It is unclear whether TEER provides the same benefit in this setting, however prior studies have shown a reduction in ventricular arrhythmias after MitraClip placement for secondary or functional MR (52, 53). Case 3 demonstrates a case in which TEER significantly reduced ventricular arrhythmias. While there are many mechanisms by which this may occur, this preliminary data suggests the need for larger studies looking at the effect of TEER on arrhythmia burden in MVP and MAD.

## Conclusions

Mitral valve prolapse and mitral annular disjunction are becoming increasingly recognized as important phenomena that can lead to malignant ventricular arrhythmias and sudden cardiac death. While much remains to be elucidated, the importance of awareness and detection of these entities is highlighted in this article, especially in patients presenting with syncope, ventricular arrhythmias, and cardiac arrest. MAD is a common finding in patients with MVP and most of the prior studies have been done in MVP populations. Given that MAD has been recently shown to be arrhythmogenic even in the absence of MVP, future research may need to focus on populations with MAD with and without MVP. Additionally, larger prospective studies are needed to optimize risk stratification and management.

## Author contributions

Both authors listed have made a substantial, direct, and intellectual contribution to the work and approved it for publication.

## Conflict of interest

The authors declare that the research was conducted in the absence of any commercial or financial relationships that could be construed as a potential conflict of interest.

## Publisher's note

All claims expressed in this article are solely those of the authors and do not necessarily represent those of their affiliated organizations, or those of the publisher, the editors and the reviewers. Any product that may be evaluated in this article, or claim that may be made by its manufacturer, is not guaranteed or endorsed by the publisher.
